# An unusual localisation of a superficial angiomyxoma

**DOI:** 10.11604/pamj.2017.28.117.13277

**Published:** 2017-10-06

**Authors:** Ben Salah Imen, Moukit Mounir

**Affiliations:** 1 Department of Obstetrics and Gynecology, Military Training Hospital Mohammed V, Rabat, Morocco

**Keywords:** Superficial angiomyxoma, cervix, surgery

## Image in medicine

A 40-year-old woman, without medical or surgical history, presented in our department for a gradually enlarged mass exteriorized from vagina (figure A). There was no history of vaginal discharge, lower abdominal pain or urinary complaints. On speculum examination, a pedunculated, non-tender, brownish and elastic tumor measuring 8 x 2 cm arising from the anterior cervix labium was noted (figure B), suggestive of a fibroepithelial polyp or a soft tissue tumors. The cervix was macroscopically normal. Under spinal anesthesia, a circular incision was made around the cervix implantation of the lesion with clear margins (about 1cm of the normal tissue) forcing excision of the mass. Histopathological examination revealed a lobulated tumor containing prominent myxoid stroma and thin walled vessels, with spindle to stellate tumor cells in higher power view; there were no atypical or mitotic figures in the tumor cells. The pathologic report was benign superficial angiomyxoma. The postoperative course was smooth and she was discharged 1 day after surgery. No recurrence was noted during a postoperative follow-up period of 2 years. Superficial Angiomyxoma is a benign mesenchymal tumor which rarely occurs in cervix, but it should be considered in differential diagnosis of cervical lesions, especially in women of reproductive age.

**Figure 1:**
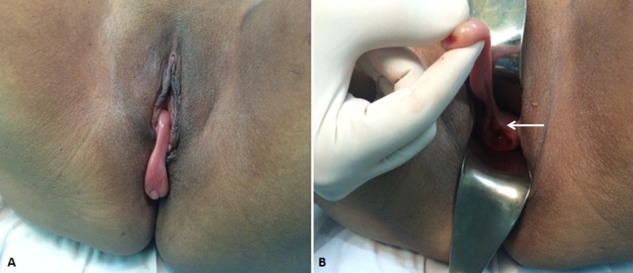
(A) gynecological examination revealing a pedunculated brownish mass exteriorized from vagina; (B) arising from the anterior cervix labium (white arrow)

